# Investigation of the Effectiveness of Disinfectants Used in Meat-Processing Facilities to Control *Clostridium sporogenes* and *Clostridioides difficile* Spores

**DOI:** 10.3390/foods10061436

**Published:** 2021-06-21

**Authors:** Siobhán McSharry, Leonard Koolman, Paul Whyte, Declan Bolton

**Affiliations:** 1 Teagasc Food Research Centre, Ashtown, 15 Dublin, Ireland; siobhan.mcsharry@teagasc.ie (S.M.); leonardkoolman87@gmail.com (L.K.); 2 School of Veterinary Medicine, University College Dublin, Belfield, 4 Dublin, Ireland; paul.whyte@ucd.ie

**Keywords:** disinfectants, sporicidal agents, spores, *Clostridium sporogenes*, *Clostridioides difficile*, D values

## Abstract

Spore-forming bacteria are a major concern for the food industry as they cause both spoilage and food safety issues. Moreover, as they are more resistant than vegetative cells, their removal from the food processing environment may be difficult to achieve. This study investigated the efficacy of the ten most commonly used disinfectant agents (assigned 1–10), used at the recommended concentrations in the meat industry, for their ability to eliminate *Clostridium sporogenes* and *Clostridioides difficile* spores. Test-tube based suspension assays suggested that disinfectants 2 (10% *v*/*v* preparation of a mixture of hydrogen peroxide (10–30%), acetic acid (1–10%) and peracetic acid (1–10%)), 7 (4% *w*/*v* preparation of a mixture of peroxymonosulphate (30–50%), sulphamic acid (1–10%) and troclosene sodium (1–10%)) and 10 (2% *v*/*v* preparation of a mixture of glutaraldehyde (10–30%), benzalkonium chloride (1–10%)) were the most effective formulations. D-values for these ranged from 2.1 to 8.4 min at 20 °C for the target spores. Based on these findings, it is recommended that these disinfectants are used to control *Clostridium* spores in the meat plant environment.

## 1. Introduction

Cleaning and disinfection are essential to prevent the food processing environment, including equipment, from acting as a source of bacterial pathogens and spoilage agents [1]. Effective disinfection (the process of cleaning to destroy bacteria) may be achieved using chemical or physical treatments [2,3,4]. While all disinfectants kill vegetative cells, those based on alcohols, phenols or quaternary ammonium compounds may not destroy bacterial spores even at high concentrations [5]. Bacterial spores are ubiquitous in meat slaughter and processing plants as they are continually introduced on contaminated hides [6,7]. Moreover, cross-contamination from contaminated equipment, such as the hide/fleece puller, knives and cutting boards is unavoidable [8].

*Clostridium sporogenes* and *Clostridioides difficile* are spore-forming anaerobic bacteria [9,10]. *C. sporogenes* are a good representative for *Clostridium* spp. in studies that evaluate disinfectants [11], including acting as a surrogate for *Clostridium botulinum* [12,13,14,15]. *Clostridioides difficile* (formerly *Clostridium difficile*; [16,17]) are Gram-positive, toxin-producing bacteria that can cause diarrhoea in humans, potentially leading to severe illness and death [18]. Although previously considered to be exclusively associated with healthcare facilities, molecular methods, such as whole genome sequencing (WGS), suggest that a significant proportion of *C. difficile* infections may be community acquired [19]. These bacteria readily colonise the intestinal tract of food animals, are shed in the faeces, and contaminate hides and fleece before transfer to carcasses and, ultimately, meat products [20,21,22]. Bouttier et al. [23] reported *C. difficile* in vacuum-packaged ground meat and recent studies in our laboratory suggest that these bacteria are common in beef, pork and lamb abattoirs.

Glutaraldehyde, formaldehyde, hydrogen peroxide, peroxy acids, as well as iodine and chlorine-based compounds, have sporicidal activity and are used individually or in combination with disinfectants [24]. Their efficacy mainly depends on the concentration of the compound and contact time, assuming the main organic debris/waste (bone dust, blood, etc.) has been removed prior to disinfection [24,25]. There are currently several different formulations that are commercially available, but no information on their relative effectiveness on which meat plant managers can make an informed decision about purchase and use.

The objective of this study was to test the sporicidal efficacy of the ten most commonly used disinfectants (at the recommended concentrations for use) marketed as sporicidal agents for use in food processing plants, including abattoirs. Further investigation would then establish the D-values for the three most effective disinfectants to establish the minimum contact times required to ensure the complete destruction of a target concentration of spores.

## 2. Materials and Methods

### 2.1. Preparation and Harvesting of Spores

#### 2.1.1. Clostridium Sporogenes

*Clostridium sporogenes* (DSM 767) was purchased as live cultures in broth from the Deutsche Sammlung von Mikroorganismen und Zellkulturen GmbH (DSMZ, Braunschweig, Germany). Exactly 100 µL of the *C. sporogenes* suspension was pipetted into deoxygenated cooked-meat medium (CMM; Oxoid Ltd., Hampshire, United Kingdom (CM0081)) and incubated anaerobically at 37 °C for 12–18 h in an anaerobic cabinet (Don Whitley, West Yorkshire, United Kingdom). Exactly 300 µL of the CMM suspension was then plated onto Campden Sporulation Agar (CSA; [26]) and anaerobically incubated at 37 °C for 12 days. In a laminar flow cabinet, 5 mL of chilled sterile distilled water (SDW) was added to the surface of the CSA plates and a sterile spreader was used to gently agitate the surface of the agar to form a spore suspension. This suspension was transferred to another CSA plate and the agitating process was repeated. The spore suspensions were then pooled to fill a 50 mL tube, vortexed and centrifuged 8–13 times at 7500× *g* at 4 °C for 15 min and resuspended with 25 mL SDW after each spin to remove cell debris and achieve the high purification of spores. The resuspension volume of SDW was diluted to achieve the required final spore concentration and the presence of spores was confirmed using the Schaeffer and Fulton’s Method [27]. The spore concentration was estimated by plating 1 mL of a heat-treated (80 °C for 10 min) suspension on deoxygenated reinforced clostridial agar (RCA; Oxoid, CM0151). Spore suspensions were placed in 1 mL aliquots and frozen at −80 °C until required for inoculum preparation.

#### 2.1.2. Clostridioides Difficile

*Clostridioides difficile*, strain D17MD99, is ribotype 126, which has been frequently recovered from carcasses, meat products and other foods and has been associated with human disease [28]. Spores were prepared and harvested as previously described [29,30]. Briefly, *C. difficile* was grown in brain heart infusion broth, supplemented with 0.1% L-cysteine and 5 mg/mL yeast extract (BHI; Oxoid, (CM1135)) agar overnight at 37 °C in an anaerobic workstation (Don Whitley). A starter culture was prepared by inoculating 1% of the overnight cultures in 2 mL BHI broth and incubated for 16 h until an optical density at 600 nm (OD_600_) of between 0.2 and 0.5 was obtained. BHI broth was inoculated with 1% of this starter culture and incubated for 5 days at 37 °C to allow for sporulation. Following sporulation, tubes containing the spore suspensions were pooled, vortexed and centrifuged 4 times at 7500× *g* at 4 °C for 10 min and the pellet was resuspended with SDW after each spin. From this culture, 1 mL was extracted, heated at 60 °C for 25 min to kill any vegetative cells present and the spores enumerated on BHI agar to estimate the final concentration (log_10_ CFU/mL). The spore suspension was also viewed under the microscope to assess purity. The inoculum was dispensed in 1 mL aliquots and frozen at −80 °C until required.

### 2.2. Preparation of Disinfectant Agents

The ten commercial disinfectants tested in these studies, chemical formulation and the concentrations used are provided in Table 1. Solutions of each agent were prepared as recommended by the manufacturer to double the required concentration. Additionally, separate tubes containing 9 mL sterile distilled water (SDW) were also prepared. All tubes were vortexed and placed in a 25 °C incubator to equilibrate overnight to eliminate any temperature variability.

### 2.3. Suspension Tests

The previously prepared 1 mL aliquots, containing *C. sporogenes* or *C. difficile* spores (10^8^–10^9^ CFU/mL), were removed from the freezer and allowed to thaw under chilled conditions. A 1 mL aliquot containing the spore suspension was transferred to 9 mL SDW and vortexed before being added to a test-tube containing 10 mL of the double strength disinfectant agent. SDW was used as the control and 4 test-tubes were prepared for each disinfectant. Each tube was thoroughly vortexed and left on the laboratory bench at a temperature of approximately 18 °C. After 20 and 60 min, 2 test-tubes for each disinfectant and the control were removed, centrifuged (at 7500× *g* at 4 °C for 10 min), the pellet resuspended in 1.5 mL of SDW and vortexed.

The spores were then resuspended in sterile Eppendorf tubes and put on a heating block (Techne Dri-Block DB-3) for 10 min at 80 °C for *C. sporogenes* or for 25 min at 60 °C for *C. difficile* to remove any vegetative cells. The ability of a given disinfectant to reduce the target spore concentration was evaluated in suspension tests in SDW and in broth RCM (reinforced clostridial medium (RCM; Oxoid, (CM0149))) or BHI broth, respectively, for *C. sporogenes* and *C. difficile* to study the efficacy of these agents in the presence of low concentrations of organic matter.

### 2.4. Calculating Decimal Reduction Times (D-Values) for Most Effective Disinfectants

D-values were obtained in broths (RCM/BHI) by removing a sample from the broth-disinfectant-spore mixture periodically (disinfectant 2 sampled at t = 0, 2, 4, 6, 8, 10, 12 min, disinfectant 7 at t = 0, 4, 8, 12, 16, 20, 24 min and disinfectant 10 were analysed at t = 0, 5, 10, 15, 20, 25, 30 min) and enumerating the surviving spores as described below. As it was essential to immediately stop the sporicidal action, the samples removed were immediately added to 10 mL Dey–Engley’s neutralising broth (D3435, Merck Life Science, Dorset, United Kingdom) and allowed stand at room temperature (approximately 18 °C), centrifuged, (7500× *g* at 4 °C for 10 min) and the pellet resuspended in 1.5 mL of SDW and vortexed. These were heat treated, as above, to ensure that the vegetative cells were destroyed.

### 2.5. Microbial Analysis

Serial dilutions of the spore suspensions were prepared in maximum recovery diluent (MRD; Oxoid, CM0733), and plated on various agars for each organism. Deoxygenated RCA was used for the enumeration of *C. sporogenes* and incubated anaerobically at 37 °C for 72 h using AnaeroGen sachets (Oxoid, AN0035) and an Anerojar (Biomerieux, 96128). *C. difficile* was enumerated on pre-reduced *C. difficile* moxalactum–norfloxacin (CDMN) agar (Oxoid, CM0601), containing CDMN supplement (Oxoid, SR0096E), together with 7% (*v*/*v*) defibrinated horse blood (Cruinn Diagnostics Ltd., Dublin, Ireland, HB034), and was incubated anaerobically at 37 °C for 48 h.

### 2.6. Statistical Analysis

The assessment for efficacy was performed in triplicate and the D-values in duplicate and repeated 3 times. Data from the experiment were analysed using a two-way ANOVA with disinfectant and treatment time as different variables. Tukey’s multiple comparisons post hoc test were performed with significance defined at *p* < 0.05. Statistical analysis was performed using GraphPad Prism 7.02 (Graphpad Software Incorporated, San Diego, CA, USA). The D-values were obtained by plotting surviving spores against time and obtaining the inverse of the slope.

## 3. Results

The sporicidal efficacy of ten different branded disinfecting agents against *C. sporogenes* and *C. difficile* spores was tested in SDW and RCM (*C. sporogenes*) or BHI (*C. difficile*) for 20 and 60 min (Table 2).

As for SDW tests, after 20 min the *C. sporogenes* spores were reduced from 7.0 log_10_ CFU/mL to between 0.5 and 4.9 log_10_ CFU/mL with similar counts achieved after 60 min. Although all disinfecting agents achieved lower counts compared to the control, disinfectants 1, 2, 3, 4, 5, 6, 7, 9 and 10 were effective, achieving significantly (*p* < 0.05) lower counts after both 20 and 60 min. The *C. sporogenes* spore counts in RCM broth ranged from 0.1 to 6.1 log_10_ CFU/mL after 20 min and from 0.1 to 5.9 log_10_ CFU/mL after 60 min. After 20 min disinfectants 2, 6 and 7 displayed the highest sporicidal activity followed by 1, 4, 9 and 10, while 3, 5 and 8 were statistically similar to the control. After 60 min exposure, disinfectant 10 joined 2, 6 and 7 as the most effective at reducing the spore concentrations. Disinfectants 1, 3, 4 and 9 also achieved significantly (*p* < 0.05) lower counts, while 5 and 8 counts were still statistically similar to the control. Thus, based on these results, disinfectants 2, 6 and 7 were the most effective sporicidal agents, while 3, 5 and 8 were the least effective.

Similar results were obtained with *C. difficile* spores in SDW, where disinfectants 2, 4, 5, 7 and 9 achieved significantly lower counts, as compared to the controls and the other disinfectants after both 20 and 60 min with counts of 0.5 log_10_ CFU/mL or less. *C. difficile* counts in solutions with 3, 6, 8 and 10 were also significantly lower than the control and 1 (which were statistically similar) but were significantly higher than 2, 4, 5, 7 and 9. When tested in BHI broth, disinfectants 2 (1.1 log_10_ CFU/mL) and 7 (2.4 log_10_ CFU/mL) achieved the lowest *C. difficile* counts after 20 min. These were joined by 10 (0.3 log_10_ CFU/mL) after 60 min with counts that were significantly lower than 6, 8 and 9 which were, in turn, significantly lower than 1, 3, 4 and 5, all of which were statistically similar to the control. Thus, disinfectants 2, 7 and 10 were considered to be the top three sporicidal agents against *C. difficile* while 1, 3, 4 and 5 were the least effective at killing these spores.

**Figure 1:**
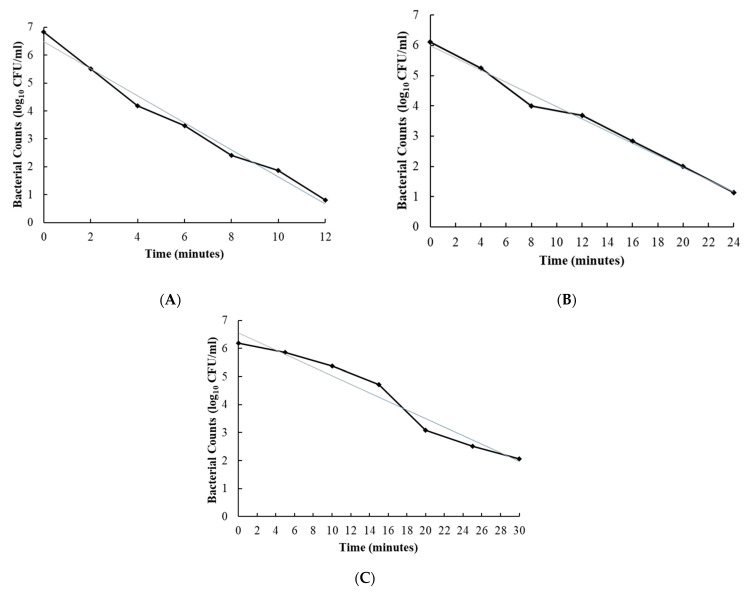
Inactivation of *Clostridium sporogenes* by disinfecting agents (Disinfectants 2 (**A**), 7 (**B**) and 10 (**C**)). Solid line represents the observed values over different time points. The straight line represents the linear regression to determine the slope, to calculate D-values.

**Figure 2:**
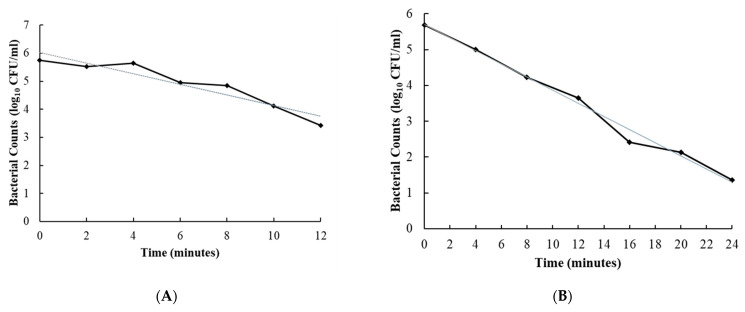

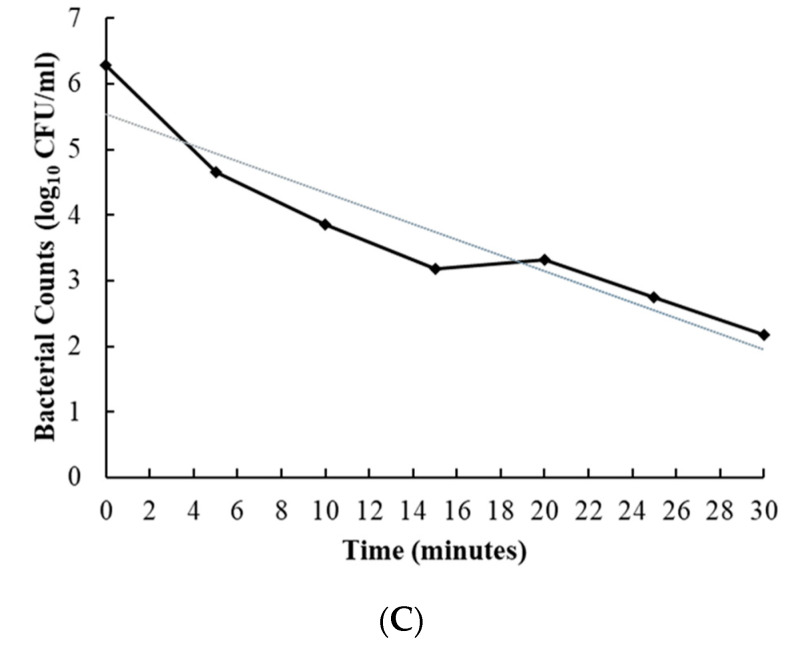
Inactivation of *Clostridioides difficile* by disinfecting agents (disinfectants 2 (**A**), 7 (**B**) and 10 (**C**)). Solid line represents the observed values over different time points. The straight line represents the linear regression to determine the slope and to calculate D values.

The D-values for disinfectants 2, 7 and 10 were 2.1, 5.0 and 6.6 min for *C. sporogenes* in RCM broth and 5.3, 5.5 and 8.4 min for *C. difficile* in BHI broth, respectively, with R^2^ values ranging from 0.90 to 0.99 (Figure 1 and Figure 2, Table 3).

## 4. Discussion

In this study, ten commercially available disinfectants that are commonly used to disinfect meat plants, including sporicidal agents, were evaluated for their efficacy at killing *C. sporogenes* and *C. difficile* spores. The most effective disinfectants contained hydrogen peroxide, peracetic acid, acetic acid, peroxymonosulphate, sulphamic acid, troclosene sodium, glutaraldehyde and/or benzalkonium chloride as these ingredients were all part of the formulations displaying the greatest sporicidal activity.

Hydrogen peroxide and peracetic acid are two of the most important peroxygens in disinfectants as they are effective against bacteria, virus, fungi and spores [4,24,31,32,33]. Both are used for industrial processes, including food plant and equipment decontamination and medical device disinfection/sterilization [34], using high concentrations (30% or greater) to ensure sporicidal properties [35]. Sattar et al. [36] reported that a 7% solution of hydrogen peroxide took up to 6 h to inactivate spores, whereas Wardle et al. [37] exposed *Bacillus* spores to 10% hydrogen peroxide and achieved complete elimination after 1 h. This agent works by removing protein from the spores’ coats [38] and producing destructive hydroxyl free radicals that can attack membrane lipids, DNA, and other essential cell components [33]. Similarly, peracetic acid denatures proteins, disrupting cell-wall permeability, and oxidizes sulfhydryl and sulphur bonds in proteins, enzymes, and other metabolites [32]. This agent is considered a more potent sporicide, compared to hydrogen peroxide, whilst being only slightly affected by the presence of organic matter [39,40], but is more corrosive than hydrogen peroxide [41]. Several studies have reported that 0.05%–1% peracetic acid inactivates bacterial spores in 15 s to 30 min using spore suspension tests [32,42,43] including inactivation of mesophilic *Clostridium* spores [44,45,46,47]. Both agents are stable in the presence of an acid [24,33] and acetic acid is often added to increase the efficacy of hydrogen peroxide and peracetic acid. Indeed, acetic acid is especially useful for this function as it is relatively nontoxic, inexpensive, and widely available [48]. Interestingly, disinfectants 2 and 3 contained the same compounds (hydrogen peroxide, peracetic acid and acetic acid) at approximately the same concentrations, but the former was the most effective sporicidal agent tested, while the latter was ineffective. This was attributed to disinfectant 2 being applied at 10% (*v*/*v*) and 3 at 2% (*v*/*v*), as recommended by the manufacturers. This highlights the importance of using an effective concentration for deactivation.

Peroxymonosulphate is an oxidizing agent, which is widely used for controlling spore-forming organisms [34] and is active in the presence of moderate organic debris. Its oxidising mechanism includes the destruction of proteins, especially disrupting the structural proteins of bacteria [49] and is ideal for use within food-processing industries and hospital environments [50,51]. Sulphamic acids are effective against bacteria, viruses and some fungi. Typically, they are coloured to facilitate an estimate of the concentration during preparation and indicate when they need to be replaced [52]. This acid is used for disinfecting surfaces, rinsing equipment and for the removal of mineral deposits, such as hard water. However, despite their efficacy, these acids are corrosive for a range of materials and must be used with care [53]. Troclosene sodium (sodium dichloroisocyanurate (NaDCC)) is a chlorine-releasing agent, primarily used for the disinfection of water [54] and commonly used as a disinfectant in health care facilities [55]. This agent gradually releases hypochlorous acid overtime, which is responsible for antimicrobial action [24,56,57].

Glutaraldehydes is an aldehyde, one of the most potent sporicidal agents and widely used for high-level disinfection of critical medical facilities [5]. This chemical inactivates spores by cross-linking outer proteins and blocking normal germination events [58]. Aqueous glutaraldehydes solutions are acidic and, generally, in this state are not sporicidal, they are only activated by the addition of alkalinizing agents [59], such as benzalkonium chloride [33]. Benzalkonium chlorides (BACs) are chemicals with widespread applications due to their broad spectrum of antimicrobial properties against bacteria, fungi, and viruses [60] and remain stable for both short- and long-term usage [61]. These chemicals are among the most common active ingredients in disinfectants [62] used in residential, industrial [63], agricultural, and clinical settings to inactive spores. March et al. [59] tested a commercially available 2.4% alkaline glutaraldehyde solution (similar concentration to disinfectant 10) as a disinfectant against *C. sporogenes* spores, and achieved a 6 log_10_ CFU/mL reduction in approximately 23 min. Rutala et al. [31] achieved complete elimination of *C. difficile* using a 2% alkaline glutaraldehyde after 10 min and concluded that *C. difficile* spores are more susceptible to inactivation by glutaraldehyde-based disinfectants than *C. sporogenes*, as was also observed in our study.

Other compounds were less effective, including alcohols C9-11, propan-2-OL, sodium hydroxide, sodium hypochlorite, iodine, didecyldimethylammonium chloride, N-(3-Aminopropyl)-N-Dodecylpropane-1,3-Diamine, alkylethersulphates, alkylamine oxide, octanoic acid, peroxyoctanoic acid, tetrasodium ethylene diamine tetraacetate, orthophosphoric acid and sulphuric acid.

Alcohols, such as alcohols C9–11, ethoxylated and propan-2-OL, are considered fast-acting disinfectants that are capable of killing most bacteria within five minutes of exposure but are ineffective against spores [35,64]. While sodium hydroxide (alkali), sodium hypochlorite and iodine (halogen) are also effective against bacteria, fungi, viruses, and mycobacterium [35,65,66] they may also have sporicidal activity, if the concentrations are sufficiently high. However, these compounds tend to be included at low concentrations as they are highly corrosive [35,66,67]. Thus disinfectants 5 and 9, containing low concentrations of these ingredients (ranging between 1–10%), were ineffective sporicidal agents.

Didecyldimethylammonium chloride (DDAC) is a typical quaternary ammonium biocide and has antimicrobial properties [68,69]. Yuan et al. [70] achieved a synergistic sporicidal effect when DDAC was combined with aldehyde and alcohol. Indeed, disinfectant 8 had this formulation but was most likely ineffective as the concentrations for use recommended by the manufacturer (1%, *v*/*v*) were too low.

Some ingredients added to disinfectants enhance the displacement of physical particles, rather than destroy microorganisms. N-(3-Aminopropyl)-N-Dodecylpropane-1, 3-Diamine, alkylethersulphates, alkylamine oxide, octanoic acid and peroxyoctanoic acid act as surfactants [71,72,73,74], whereby they loosen particles (such as dirt, clay, and oil) from surfaces [75,76]. Similarly, acidic disinfectants, including tetrasodium ethylene diamine tetra-acetate, orthophosphoric and sulphuric acid are added to remove calcium salts and metal oxides [76], which leads to sedimentation and incrustation in containers, pipes and nozzles [77]. Furthermore, acids can change the pH of disinfectants thereby increasing their antimicrobial activity [78].

Overall, there are many different available ingredients that may be included in disinfectant formulations. Thus, it is essential to understand the mechanism of action of each agent and potential synergy to allow for an informed decision on the most effective formulations. When evaluating the effectiveness of cleaning products, it is recognized that for many disinfectants organic matter reduces activity by reacting with the disinfectant or completely preventing the disinfectant from accessing its microbial target [79,80,81,82]. Moreover, not all chemicals can destroy spores, while other may be highly corrosive or present health and safety issues which prevent their application. Although not an objective of our research, this study also observed that *C. sporogenes* are less resistant to disinfectants as compared to *C. difficile* spores. *C. sporogenes* achieved D-values of between 2.06 and 6.56 min for the three most effective sporicidal agents (2, 7 and 10), while the corresponding values for *C. difficile* spores ranged from 5.28 to 8.38 min. Differences in spore susceptibility to a given chemical agent may be due to differences in the composition and structure of the spore coat, which is usually composed of small acid-soluble spore proteins and an inner membrane, which is usually immobile and impermeable [83,84]. Regardless, based on our findings, the recommended disinfecting time of 20 min for application in the meat industry would be insufficient to eliminate any spores, even if present in high concentrations. A potential limitation of this study is the variance in concentrations of the active compounds in each of the formulations tested in this study (Table 1). This batch-to-batch variation occurs during manufacturing [85] and, thus, the experimental design reflects what happens in the real world under commercial conditions of application. Moreover, as the neat product is diluted to at least 1 in 10 (10%), but may be as high as 1 in 100 (1%), differences in the final concentrations of the active ingredients are usually negligible. There are exceptions to this; for example, for disinfectant 2, which has the highest potential range of any active ingredient (hydrogen peroxide, 1% to 3%) in the final solution. Further research would be required to determine if this significantly affects the efficacy of this treatment.

## 5. Conclusions

This study explored the effectiveness of ten commercially available disinfectants, commonly used as sporicidal agents in the meat and other food processing industries. Disinfectants containing sporicidal agents, such as hydrogen peroxide, peracetic acid, peroxymonosulphate and glutaraldehyde were the most effective at inactivating spores when these chemicals were at sufficiently high concentrations in the product formulation. These disinfectants should, therefore, be used at the manufacturer-recommended concentrations to ensure the effective destruction of *Clostridium* spp. from the production environment. However, further studies are undoubtedly necessary to examine these sporicidal agents within environments containing natural organic matter (blood, fat, trimmings) within meat processing plants to get a more accurate conclusion for these agents.

## Figures and Tables

**Table 1:** Disinfectants used in the study.

Disinfectant	Chemical Composition ^1^	Recommended Concentration
1	N-(3-Aminopropyl)-N-Dodecylpropane-1,3-Diamine (1–10%), Alcohols, C9-11, Ethoxylated (1–10%), Tetrasodium Ethylene Diamine Tetraacetate (1–10%), Propan-2-OL (1–10%)	1%
2	Hydrogen Peroxide (10–30%), Acetic acid (1–10%), peracetic acid (1–10%)	10%
3	Hydrogen peroxide (≥ 25 ≤ 30%), Acetic acid (≥ 5 ≤ 10%), peracetic acid (≥ 2.5 ≤ 5%)	2%
4	Acetic acid (10 ≤ 25%), Hydrogen Peroxide (5 ≤ 8%), Alkylethersulphates (<10%), Peracetic acid (1 ≤ 5%), Octanoic acid (1 ≤ 5%), Peroxyoctanoic acid (<1%)	1.10%
5	Sodium hypochlorite (≥ 5.2 ≤ 10%), Sodium hydroxide (≥ 5 ≤ 10%), Alkylamine oxide (≥ 3 ≤ 5 %)	5%
6	Ethylenediamine tetraacetate (≥ 10 ≤ 20%), Benzalkonium chloride (≥ 5 ≤ 10%), Isotridecanol, ethoxylated (≥ 1 ≤ 2.5%), Didecyl Dimethyl Ammonium Chloride (≥ 1 ≤ 2.5%), sodium hydroxide (≥ 0.25 ≤ 0.5%), propan-2-ol (≥ 0.1 ≤ 0.25%)	1%
7	Peroxymonosulphate (30–50%), Sulphamic Acid (1–10%), Troclosene Sodium (1–10%)	4%
8	Alkyl dimethyl benzyl ammonium chloride (15–30%), Didecyldimethylammonium chloride (5–15%), Glutaraldehyde (5–15%), Propan-2-ol (5–15%)	1%
9	Alcohols, C9-11, Ethoxylated (10–30%), Orthophosphoric acid (10–30%), Sulphuric acid (1–10%), Iodine (1–10%)	2%
10	Glutaraldehyde (10–30%), Benzalkonium Chloride (1–10%)	2%

^1^ Composition taken from the material safety data sheet (MSDS).

**Table 2:** The spore counts (log_10_ CFU/mL) after treatment with the different disinfectants for 20 and 60 min.

	*C. sporogenes* Spores								*C. difficile* Spores							
	SDW				RCM Broth				SDW				BHI Broth			
	20 Min		60 Min		20 Min		60 Min		20 Min		60 Min		20 Min		60 Min	
	C ^1^	SE ^2^	C	SE	C	SE	C	SE	C	SE	C	SE	C	SE	C	SE
Control ^3^	7.0 ^a^	0.10	7.0 ^a^	0.10	6.2 ^a^	0.25	6.2 ^a^	0.25	6.4 ^a^	0.07	6.4 ^a^	0.07	6.0 ^a^	0.05	6.0 ^a^	0.05
1	2.3 ^ce^	0.13	2.4 ^cd^	0.30	2.5 ^cd^	0.10	2.2 ^cd^	0.08	5.3 ^ab^	0.08	5.3 ^ab^	0.12	4.7 ^ab^	0.34	4.6 ^bc^	0.40
2	0.5 ^e^	0.43	0.1 ^d^	0.00	0.6 ^f^	0.28	0.2 ^e^	0.10	0.1 ^e^	0.00	0.1 ^e^	0.00	1.1 ^e^	0.14	0.1 ^d^	0.00
3	0.7 ^e^	0.68	0.5 ^d^	0.41	4.8 ^ab^	0.32	3.3 ^bc^	0.14	4.1 ^bc^	0.27	3.6 ^c^	0.45	5.9 ^a^	0.16	5.8 ^ab^	0.23
4	0.6 ^e^	0.55	0.3 ^d^	0.24	3.9 ^bc^	0.23	3.1 ^c^	0.81	0.5 ^e^	0.28	0.3 ^e^	0.16	5.8 ^a^	0.22	5.9 ^ab^	0.18
5	0.5 ^e^	0.34	0.6 ^cd^	0.33	6.1 ^a^	0.41	5.9 ^a^	0.37	0.1 ^e^	0.00	0.1 ^e^	0.00	6.0 ^a^	0.17	5.9 ^ab^	0.24
6	2.5 ^ce^	0.69	2.0 ^cd^	0.45	1.4 ^df^	0.33	0.6 ^de^	0.39	3.2 ^cd^	0.64	3.9 ^c^	0.15	4.4 ^bc^	0.22	4.1 ^c^	0.43
7	1.2 ^e^	0.81	0.4 ^d^	0.38	1.7 ^dfe^	0.21	0.1 ^e^	0.00	0.2 ^e^	0.10	0.1 ^e^	0.00	2.4 ^de^	0.21	0.2 ^d^	0.10
8	4.9 ^ab^	0.40	5.0 ^ab^	0.37	4.8 ^ab^	0.15	4.9 ^ab^	0.19	4.5 ^b^	0.06	4.6 ^bc^	0.19	4.2 ^bf^	0.42	4.2 ^c^	0.51
9	1.5 ^de^	0.73	0.5 ^d^	0.27	3.0 ^bce^	0.61	2.7 ^c^	0.81	0.3 ^e^	0.27	0.2 ^e^	0.10	3.5 ^bd^	0.26	3.8 ^c^	0.56
10	3.8 ^bcd^	0.86	2.8 ^bc^	0.69	2.8 ^ce^	0.23	0.8 ^de^	0.49	2.4 ^d^	0.40	2.2 ^d^	0.49	3.1 ^cdf^	0.11	0.3 ^d^	0.27

^1^ C = spore count; ^2^ SE = standard error; ^3^ Different treatments. Numbers that have different letters in the same column (a,b,c,d,e,f) indicate significance at *p* < 0.05.

**Table 3:** Calculated D values for *Clostridium sporogenes* and *Clostridioides difficile* spores treated with most effective disinfectants (2, 7 and 10).

Disinfectant	Spores	D Value (Minutes)	SE ^1^
2	*C. sporogenes*	2.1	±0.03
	*C. difficile*	5.3	±0.02
7	*C. sporogenes*	5.0	±0.02
	*C. difficile*	5.5	±0.01
10	*C. sporogenes*	6.6	±0.01
	*C. difficile*	8.4	±0.01

^1^ SE = standard error of the slope.

## Data Availability

Not applicable.
